# In-hospital mortality of older patients with COVID-19 throughout the epidemic waves in the great Paris area: a multicenter cohort study

**DOI:** 10.1186/s12877-023-04236-y

**Published:** 2023-09-18

**Authors:** Sara Thietart, Antoine Rozes, Florence Tubach, Stéphane Marot, Anne-Geneviève Marcelin, Mathieu Raux, Hélène Vallet, Bruno Riou, Jacques Boddaert, Lorène Zerah

**Affiliations:** 1 Sorbonne Université, Assistance Publique-Hôpitaux de Paris (AP-HP), Hôpital Pitié- Salpêtrière, Département de Gériatrie, Paris, France; 2 Assistance Publique-Hôpitaux de Paris (AP-HP), Hôpital Pitié Salpêtrière, Centre de Pharmacoépidémiologie (Cephepi), Unité de Recherche Clinique PSL-CFX, CIC-1901, Paris, F75013 France; 3 Sorbonne Université, INSERM, Institut Pierre Louis d’Épidémiologie et de Santé Publique, Assistance Publique-Hôpitaux de Paris (AP-HP), Hôpital Pitié-Salpêtrière, Département de Santé Publique, Centre de Pharmacoépidémiologie (Cephepi), Unité de Recherche Clinique PSL-CFX, CIC-1901, Paris, F75013 France; 4 Sorbonne Université, INSERM, Institut Pierre Louis d’Épidémiologie et de Santé Publique, Assistance Publique-Hôpitaux de Paris (AP-HP), Hôpital Pitié-Salpêtrière, Département de Virologie, Paris, France; 5 Sorbonne Université, INSERM, UMRS1158, AP-HP, Hôpital Pitié-Salpêtrière, Département d’Anesthésie Réanimation, Paris, France; 6 Sorbonne Université, INSERM, Centre d’Immunologie et des Maladies Infectieuses (Cimi-Paris), AP-HP, Hôpital Saint Antoine, Département de Gériatrie, Paris, France; 7 Sorbonne Université, UMRS INSERM 1166, IHU ICAN, AP-HP, Hôpital Pitié- Salpêtrière, Département des Urgences, Paris, France; 8 Sorbonne Université, INSERM, Centre d’Immunologie et des Maladies Infectieuses (Cimi-Paris), Assistance Publique-Hôpitaux de Paris (AP-HP), Hôpital Pitié-Salpêtrière, Département de Gériatrie, Paris, France; 9 Sorbonne Université, INSERM, Institut Pierre Louis d’Épidémiologie et de Santé Publique, Assistance Publique-Hôpitaux de Paris (AP-HP), Hôpital Pitié-Salpêtrière, Département de Gériatrie, Paris, France

**Keywords:** COVID-19, In-hospital mortality, Geriatric, Waves, Omicron

## Abstract

**Background:**

Mortality is high in older patients hospitalized with COVID-19. Previous studies observed lower mortality during the Omicron wave, yet no data is available on older patients. The objective was to compare in-hospital mortality between the Omicron and previous waves in older patients hospitalized with COVID-19.

**Methods:**

This retrospective observational multicenter cohort study used the Greater Paris University Hospitals Group’s data warehouse (38 hospitals). Patients aged ≥ 75 years with a confirmed COVID-19 diagnosis and hospitalized from March 2020 to January 2022 were included. The study period was divided into five waves. The fifth wave (January 1st to 31st 2022) was considered as the Omicron wave as it was the predominant variant (≥ 50%), and was compared with waves 1 (March-July 2020), 2 (August-December 2020), 3 (January-June 2021) and 4 (July-December 2021). Primary outcome was in-hospital mortality. Secondary outcome was occurrence of ICU admission or in-hospital death. Multivariate logistic regression was performed, with a sensitivity analysis according to variant type.

**Results:**

Of the 195,084 patients hospitalized with COVID-19, 19,909 patients aged ≥ 75 years were included (median age 85 [IQR 79–90] years, 53% women). Overall in-hospital mortality was 4,337 (22%), reaching 345 (17%) during wave 5. Waves 1 and 3 were significantly associated with increased in-hospital mortality in comparison with wave 5 (adjusted Odds Ratios aOR 1.42 [95%CI 1.21–1.66] and 1.56 [95%CI 1.33–1.83] respectively). Waves 1 to 3 were associated with an increased risk of occurrence of ICU admission or in-hospital death in comparison with wave 5: aOR 1.29 [95% CI 1.12 to 1.49] for wave 1, aOR 1.25 [95% CI 1.08 to 1.45] for wave 2 and aOR 1.56 [95% CI 1.36 to 1.79] for wave 3. Sensitivity analysis found that Omicron variant was associated with decreased mortality, in comparison with previous variants.

**Conclusions:**

Mortality was lower during the 5th Omicron wave in the older population, but remained high, implying that this variant could be considered as “milder” but not “mild”. This persistently high mortality during the 5th Omicron wave highlights the importance of including older patients in clinical trials to confirm the benefit/risk balance of COVID-19 treatments in this fragile population.

**Supplementary Information:**

The online version contains supplementary material available at 10.1186/s12877-023-04236-y.

## Background

Older patients with coronavirus disease 19 (COVID-19) have a higher mortality rate than younger patients [1, 2]. In-hospital mortality in geriatric COVID-19 wards in the Paris region reached 31% during the first wave [3]. Older patients are at higher risk of respiratory distress syndrome, but are also burdened by higher comorbidities, loss of functional status and drug related events which could negatively affect outcomes [3–6].

A SARS-CoV-2 (severe acute respiratory syndrome coronavirus 2) variant of concern, Omicron (B.1.1.529), arrived in France early December 2021 and rapidly became the predominant variant, being responsible for a 5th wave of COVID-19 in France [7, 8]. A French study recently described that in-hospital mortality was lower when patients were infected with the Omicron variant (11%), than with the Delta variant (17%), but median age was 58 years [7]. A multicenter South African study observed a decreased in-hospital mortality down to 3% in the Omicron wave, instead of 29% in the previous wave [9]. Median age in this study was only 34 years during the Omicron wave. Another South African study on patients infected with the Omicron variant had reduced odds of hospitalization and severe disease than those infected with other variants, but the proportion of patients aged ≥ 60 years in this study was only 7% [2]. A retrospective study of patients aged 73 years observed in 60 patients hospitalized during the Omicron wave that in-hospital mortality was 6.7% [10]. A monocentric study observed in patients with a median age 74 years, observed an in-hospital mortality of 11.6% during the Omicron wave, lower than the previous wave (17.6%) [11]. Data on outcomes of patients aged ≥ 75 years hospitalized with COVID-19 throughout the epidemic waves is scarce.

Due to a higher prevalence of comorbidities, loss of functional status, and an unclear benefit/risk balance of COVID-19 treatment, mortality of older patients during the Omicron wave could be higher than what has been previously published in the younger population. A prospective multicentre study on 2,625 patients aged ≥ 75 years hospitalized in intensive care units (ICU) for COVID-19 found an increased mortality rate during the second wave, comparatively with the first one, although intensivist had acquired more experience during the second wave [12]. Progress could be made in COVID-19 management in the older population. In addition, the older population is under-represented in clinical trials on COVID-19, and the benefit/risk balance of COVID-19 treatments awaits to be confirmed [13]. We hypothesize that mortality of older patients with COVID-19 remains high throughout the different pandemic waves.

The objective of this study was to compare in-hospital mortality between the 5th Omicron wave and previous waves in older patients hospitalized with COVID-19.

## Methods

Our manuscript complies with the Reporting of studies Conducted using Observational Routinely collected health Data (RECORD) statement (Table S1) [14]. The study was approved by the Clinical Data Warehouse of Greater Paris University Hospitals’ Scientific and Ethics Committee (IRB00011591, authorization n° CSE-EDS n°22 − 05).

### Study design, setting and data source

We performed a primary data analysis on a retrospective observational multicenter cohort within 39 hospitals of the Greater Paris University Hospitals Group’s data warehouse (Entrepôt des Données de Santé de l’Assistance Publique Hôpitaux de Paris (AP-HP), France) [15], i.e. data prospectively collected in the electronic health records of all hospitalized patients from the Greater Paris University Hospitals Group. Because of interoperability issues, data from the Georges Pompidou European hospital, a hospital in the greater Paris area, are missing.

To date, this data warehouse includes anonymous recorded data on the demographic characteristics of hospitalized patients such as date of birth, sex, vital status and date of death. It also contains, for each hospitalization, the hospital discharge summary (principal and related diagnoses coded according to the International Classification of Diseases 10th edition ICD-10), medical procedures and administered drugs (Anatomical Therapeutic Chemical (ATC) codes), comorbidities of patients from medical records and ICD-10 codes, and laboratory results. Data extraction was performed the 15th of April 2022.

### Study population, follow-up and COVID-19 waves

The study cohort comprised all adults aged 75 years or over, hospitalized in one of the 38 participating hospitals between 1st of March 2020 to 31st of January 2022, with a confirmed COVID-19 diagnosis (hospital stay with an ICD-10 code for COVID-19: U07.1 except U0713, U10.9 and/or at least one SARS-CoV-2 positive RT-PCR). If a patient was hospitalized more than once for COVID-19, only the first hospitalization at AP-HP was included for analysis. Patients were included the day of admission, and followed-up until discharge, death or end of study period (15th of April 2022). Data extraction took place 10 weeks after the last patient inclusion in order to accurately collect data on mortality [12]. One hospitalization could include transfers in different acute care departments (intensive care units, acute medical wards, intermediate care units).

According to the French epidemiological data on SARS-CoV-2 circulation in the Greater Paris area [8] and by variant dominance, the overall study period was divided into 5 COVID-19 waves, defined as follows: first wave, from March to July 2020; second, from August to December 2020; third, from January to June 2021; fourth, from July to December 2021; fifth, from 1st to 31st of January 2022. The fifth wave was considered as the Omicron wave as it was the predominant variant (≥ 50% of variant determination).

### Outcomes

Primary outcome was in-hospital mortality. Secondary outcome was the composite outcome occurrence of intensive care unit (ICU) admission or in-hospital death.

### Covariates

The following data were extracted from the AP-HP data warehouse: demographic data (age, sex), comorbidities, including the Charlson score [16], hospital wards, baseline biological values (lymphocyte count, C-reactive protein (CRP), RT-PCR positivity and, when available, SARS-CoV-2 variant), complications during hospitalization (respiratory failure, use of invasive ventilation, major bleeding, thromboembolic disease, major adverse cardiovascular events (MACE)) and medications used during hospitalization. Vaccination status was not available, and extraction of texts from medical records on a randomly selected sample could not give reliable results. The list of codes is specified in the Table S2.

### Statistical analysis

Data are presented as numbers (percentages) for categorical variables, mean (standard deviation) and median [first to third quartiles, interquartile range, IQR] for quantitative variables with normal and non-normal distribution, respectively. Normality was assessed with the Kolmogorov-Smirnov test and a graphical representation of the distribution. Chi-squared test or Fisher test were used for categorical variables and Kruskal Wallis test was used for continuous variables.

Association analyses were conducted using logistic regression models using 5th Omicron wave as reference group. Adjusted odds ratios (aORs) with 95% confidence intervals (CIs) were calculated. All variables with a *P*-value < 0.20 on univariate analysis were included in the multivariate analysis. Age, sex, comorbidities (coronary heart disease, non-valvular atrial fibrillation, organ transplantation, cancer or hematologic malignancy evolving since 5 years or less, chronic kidney disease, dementia, chronic obstructive pulmonary disease and diabetes), baseline CRP and baseline lymphocyte count were evaluated as potential confounders. Continuous variables were dichotomized by receiver operating characteristic curve analysis to determine the best threshold for in-hospital mortality (maximization of the Youden index). A pre-specified sensitivity analysis included only individuals with available information on SARS-CoV-2 variant. Association analyses were conducted using logistic regression models using the Omicron variant as the reference group, using the same method as for primary outcome.

Statistical analyses were performed using R software (version 4.1.2.). Bilateral alpha risk was set at 0.05.

## Results

### Patient characteristics and variant determination

**Fig. 1:**
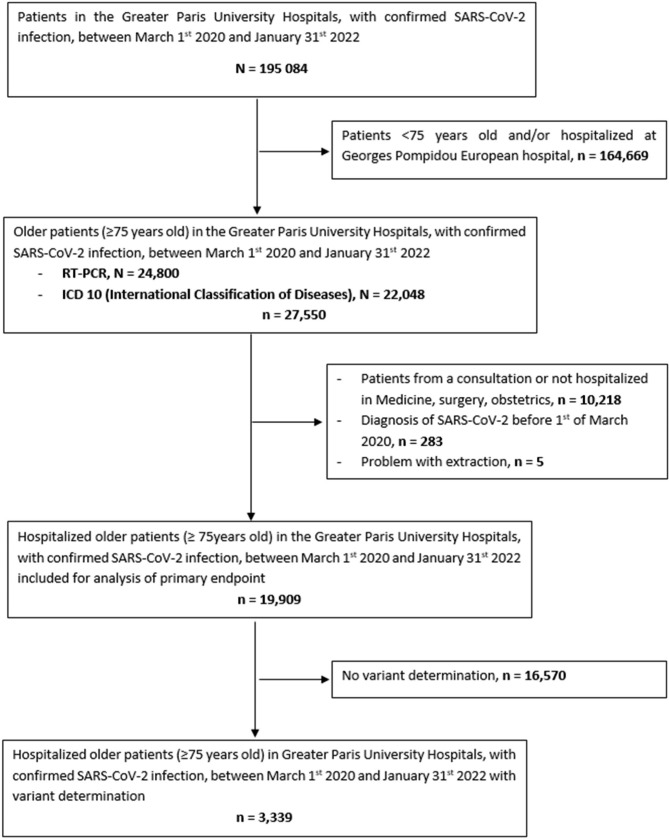
Flow chart

**Fig. 2:**
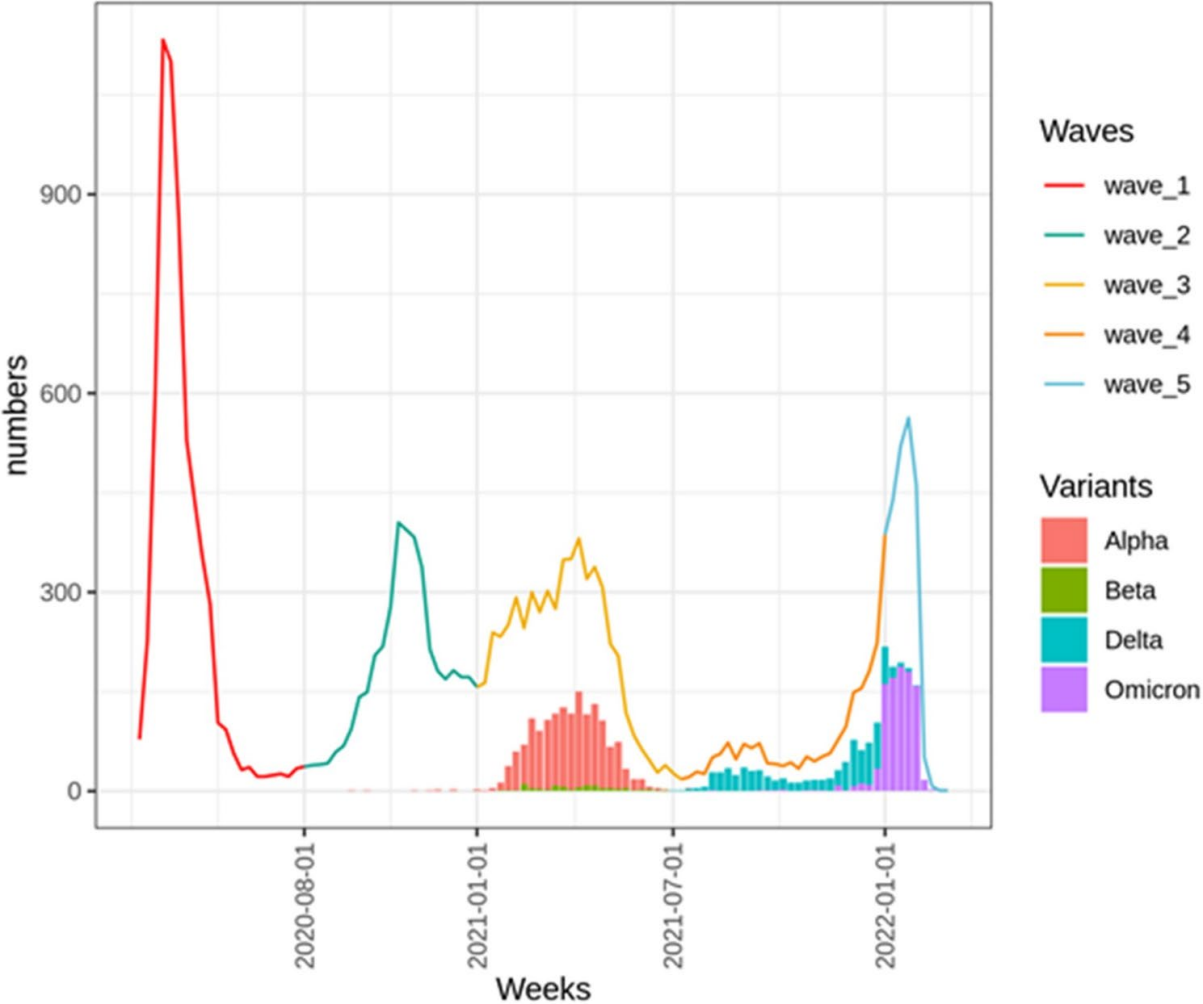
Number of patients ≥ 75 years hospitalized with COVID-19 according to COVID-19 waves and SARS-CoV-2 variants. 1st wave between March 1st 2020 and July 31st 2020, 2nd wave between August 1st 2020 and December 31st 2020, 3rd wave between January 1st 2021 and June 30th 2021, 4th wave between July 1st 2021 and December 31st 2021, and 5th Omicron wave between January 1st and 31st 2022. Solid histograms represent data with variants

Among the 195,084 patients hospitalized with COVID-19, 19,909 patients aged 75 years or over were included, with 3,339 variant determination (Fig. 1). Median age was 85 years [IQR 79–90], with 10,461 women (53%), and a median Charlson score of 3 [IQR 2–5]. Median hospitalization duration was 10.0 days (IQR 3.9–18.8). Other characteristics are described in Table 1. Figure 2 shows the number of patients hospitalized according to each wave and variant type.

**Table 1:** Characteristics of older patients admitted with COVID-19 during the 5 epidemic waves

	Total N = 19,909	Wave 1^a^ N = 6,114	Wave 2 ^a^ N = 4,070	Wave 3 ^a^ N = 5,485	Wave 4 ^a^ N = 2,168	Wave 5 ^a^ N = 2,072	Global *P-* value^b^
**Age, median (IQR)**	85 (79–90)	85 (80–91)*	84 (79–90)	84 (79–90)	84 (79–90)	85 (80–90)	**< 0.001**
**Female sex, n (%)**	10,461 (53)	3,320 (54)	2,038 (50)	2,957 (54)	1,085 (50)	1,061 (51)	**< 0.001**
**Comorbidities at baseline, n (%)**							
Dementia	6,562 (33)	2,255 (37)*	1,370 (34)	1,617 (29)	663 (31)	657 (32)	**< 0.001**
Atrial fibrillation	5,453 (27)	1,640 (27)	1,094 (27)	1,532 (28)	620 (29)	567 (27)	0.41
Diabetes	5,626 (28)	1,631 (27)	1,213 (30)	1,597 (29)	597 (28)	588 (28)	**0.005**
Hypertension	11,328 (57)	3,459 (57)*	2,365 (58)*	3,164 (58)*	1,224 (56)	1,116 (54)	**0.02**
Chronic kidney disease	5,263 (26)	1,597 (26)	1,118 (27)	1,454 (27)	518 (24)*	576 (28)	**0.02**
Coronary artery disease	3,851 (19)	1,172 (19)	811 (20)	1,023 (19)	408 (19)	437 (21)	0.12
Heart failure	5,516 (28)	1,678 (27)	1,124 (28)	1,556 (28)	573 (26)	585 (28)	0.48
COPD	2,562 (13)	787 (13)	528 (13)	670 (12)	294 (14)	283 (14)	0.38
Any tumor (including lymphoma, leukemia)	4,643 (23)	1,343 (22)*	913 (22)*	1,298 (34)*	549 (25)	540 (26)	**< 0.001**
Organ transplantation	235 (1)	67 (1)	35 (0.9)	71 (1)	30 (1)	32 (2)	0.10
Systemic auto-immune diseases	1,000 (5)	255 (4)*	217 (5)	278 (5)*	116 (5)	134 (6)	**< 0.001**
Charlson Index, median (IQR)	3 [2 - 5]	3 [2 - 5]	3 [2 - 5]	3 [2 - 5]	3 [2 - 5]	3 [2 - 5]	0.70
*Missing values*	*4197*	*1362*	*3259*	*4292*	*1706*	*1703*	
**SARS-CoV2 variant determination**^**c**^, **n (%)**							
Alpha	1,502 (45)	NA	10 (100)	1,491 (94)	1 (0)	0 (0)	
Beta	96 (3)	NA	NA	96 (6)	0 (0)	0 (0)	
Delta	777 (23)	NA	NA	1 (0)	745 (76)	31 (4)	
Omicron	964 (29)	NA	NA	0 (0)	237 (24)	727 (96)	
**Biological data at baseline**^**d**^, **n (%)**							
Lymphocyte count <0.81 10^9^/L	6,114 (43)	1,730 (39)	1,275 (42)*	1,861 (44)*	657 (42)	591 (39)	**< 0.001**
*Missing values*	*5,581*	*1692*	*1024*	*1241*	*584*	*1040*	
CRP at baseline ≥65 mg/L	7,121 (44)	2,299 (48)	1,474 (43)	2,003 (43)	731 (41)	614 (37)	**< 0.001**
*Missing values*	*3,640*	*1320*	*659*	*840*	*403*	*418*	
**Treatments, n (%)**							
Glucocorticoids	4,936 (25)	522 (8)*	1,198 (19)*	1,963 (36)*	695 (32)*	558 (27)	**< 0.001**
Tocilizumab	465 (2)	38 (0.6)*	35 (0.9)*	211 (4)*	124 (6)*	57 (3)	**< 0.001**

*Notes*: Missing values are detailed only when they exist. All codes for comorbidities and treatments can be found in the supplement. ^a^ 1st wave between March 1st 2020 and July 31st 2020, 2nd wave between August 1st 2020 and December 31st 2020, 3rd wave between January 1st 2021 and June 30th 2021, 4th wave between July 1st 2021 and December 31st 2021, and 5th Omicron wave between January 1st and 31st 2022. ^b^ Chi-squared test or Fisher’s exact test was used for categorical variables and Kruskal Wallis test was used for continuous variables. ^c^ Variant determination was performed on 3,339 patients. ^d^ Continuous variables were dichotomized by receiver operating characteristic curve analysis to determine the best threshold for in-hospital mortality (maximization of the Youden index). * *P* value < 0.05 versus wave 5 (reference)

Abbreviations: COPD: Chronic obstructive pulmonary disease, CRP: C-reactive protein; IQR: interquartile range; NA: not available

Variant type was available for 3,339 patients, with 1,502 (45%) being Alpha, 96 (3%) Beta, 777 (23%) Delta and 964 (29%) Omicron variant. Table 1 shows the numbers of patients with each variant type during the 5 waves. Patient characteristics according to variant type are described in Table S3.

### In-hospital mortality

Overall in-hospital mortality was 4,337 (22%), with 345 deaths (17%) during the 5th Omicron wave (Table 2). In multivariate analysis, waves 1 and 3 were significantly associated with increased in-hospital mortality in comparison with wave 5: aOR 1.42 [95% CI 1.21 to 1.66] and 1.56 [95% CI 1.33 to 1.83], respectively (Tables 2 and 3).

**Table 2:** Outcomes of older patients admitted with COVID-19 during the 5 epidemic waves (univariate and multivariate analyses)

	Total N = 19,909	Wave 1^a^ N = 6,114	Wave 2* N = 4,070	Wave 3* N = 5,485	Wave 4* N = 2,168	Wave 5* N = 2,072	Global *P*-value (univariate analysis) ^b^
Primary endpoint: in-hospital mortality							
n (%)	4,337 (22)	1,449 (24)*	805 (20)*	1,329 (24)*	409 (19)	345 (17)	**< 0.001**
aOR		1.42	1.15	1.56	1.13	1 [Ref]	
[95% CI]^c^		[1.21–1.66]	[0.97–1.37]	[1.33–1.83]	[0.92–1.36]		
**Secondary endpoint: ICU admission or in-hospital mortality**							
aOR		1.29	1.25	1.56	1.17	1 [Ref]	
[95% CI]^d^		[1.12–1.49]	[1.08–1.45]	[1.36–1.79]	[0.98–1.38]		
**Other outcomes, n (%)**							
ICU admission	2,733 (14)	655 (11)	662 (16)*	891 (16)*	299 (14)*	226 (11)	**< 0.001**
Invasive ventilation	504 (3)	129 (2)	144 (4)*	152 (3)*	50 (2)	29 (1)	**< 0.001**
Respiratory failure	4,977 (25)	1,411 (23)*	1,050 (26)*	1,652 (30)*	549 (25)*	315 (15)	**< 0.001**
Major bleeding	1,894 (9)	477 (8)*	358 (9)*	553 (10)*	259 (12)	247 (12)	**< 0.001**
Thromboembolic disease	1,006 (5)	254 (4)	189 (5)	343 (6)*	132 (6)*	88 (4)	**< 0.001**
MACE	836 (4)	216 (4) *	155 (4) *	233 (4) *	104 (5)	128 (6)	**< 0.001**

*Notes*: Missing values are detailed only when they exist. All codes for comorbidities and treatments can be found in the supplement. ^a^ 1st wave between March 1st 2020 and July 31st 2020, 2nd wave between August 1st 2020 and December 31st 2020, 3rd wave between January 1st 2021 and June 30th 2021, 4th wave between July 1st 2021 and December 31st 2021, and 5th Omicron wave between January 1st and 31st 2022. ^b^ Chi-squared test was used. ^c^ Multivariate analysis (adjusted OR, 95%CI) for primary outcome adjusted on: gender, age, coronary heart disease, non-valvular atrial fibrillation, organ transplantation, tumor, chronic kidney disease, dementia, chronic obstructive pulmonary disease, diabetes, baseline C-reactive protein, baseline lymphocyte count. N = 14,108; C-index 0.68 [95% CI 0.67 to 0.69]; AIC, 14,137. ^d^ Multivariate analysis (adjusted OR, 95%CI) for secondary outcome adjusted on: gender, age, coronary heart disease, non-valvular atrial fibrillation, organ transplantation, tumor, chronic kidney disease, dementia, chronic obstructive pulmonary disease, diabetes, baseline C-reactive protein, baseline lymphocyte count. N = 14,108; C-index 0.67 [95% CI 0.66 to 0.68], AIC, 16,724. * *P* value < 0.05 versus wave 5 (reference)

Abbreviations: aOR: adjusted odds ratio, CI: confidence interval, ICU: Intensive Care unit, MACE (major adverse cardiovascular events): stroke, myocardial infarction, systemic arterial embolism

**Table 3:** Multivariate analysis of risk of in-hospital mortality in hospitalized older patients with COVID-19

Variables	OR (95% CI)^a^	*P*-value
**Sex, reference value = Female**		
Gender = Male	1.46 (1.34–1.59)	< 0.001
**Age, reference value = 75–84**		
Age: 85–94 Age: ≥ 95	1.30 (1.19–1.42) 1.39 (1.18–1.63)	< 0.001 < 0.001
**COVID-19 waves, reference value = wave 5**		
Wave 1 Wave 2 Wave 3 Wave 4	1.42 (1.21–1.66) 1.15 (0.97–1.37) 1.56 (1.33–1.83) 1.13 (0.92–1.36)	**< 0.001** 0.21 **< 0.001** 0.23
**Coronary heart disease, reference value = no**		
Coronary heart disease = yes	1.00 (0.90–1.11)	0.96
**Non-valvular atrial fibrillation, reference value = no**		
Non-valvular atrial fibrillation = yes	1.11 (1.01–1.22)	**0.003**
**Organ transplantation, reference value = no**		
Organ transplantation = yes	1.34 (0.94–1.88)	0.10
**Tumor, reference value = no**		
Tumor = yes	1.05 (0.95–1.15)	0.34
**Chronic kidney disease, reference value = no**		
Chronic kidney disease = yes	1.10 (0.99–1.21)	0.07
**Dementia, reference value = no**		
Dementia = yes	0.83 (0.75–0.91)	**<0.001**
**COPD, reference value = no**		
COPD = yes	0.97 (0.86–1.09)	0.64
**Diabetes, reference value = no**		
Diabetes = yes	0.98 (0.89–1.08)	0.67
**CRP at baseline, reference value < 65 mg/L**		
CRP at baseline ≥ 65 mg/L = yes	2.57 (2.36–2.80)	**< 0.001**
**Lymphocytes count at baseline, reference value ≥ 0.81 10** ^**9**^ **/L**		
Lymphocyte count at baseline < 0.81 10^9^/L = yes	1.58 (1.46–1.72)	**< 0.001**

Notes: ^a^ N = 14,108; C-index 0.68 [95% CI, 0.67 to 0.69]; AIC, 14,137

Abbreviations: AIC: Akaike’s criterion; COPD: Chronic obstructive pulmonary disease, CRP: C-reactive protein, OR, odds ratio; CI, confidence interval

Sensitivity analysis performed on 3,048 patients found an increased in-hospital mortality with the Alpha (aOR 1.90 [95%CI 1.52 to 2.38]), Beta (aOR 2.10 [95%CI 1.28 to 3.38]) and Delta variants (aOR 1.77 [95CI 1.37 to 2.29]), compared with that of the Omicron variant (Table S4).

### Intensive care unit admission

Overall ICU admission was 2,733 patients (14%), with 226 admissions (11%) during the 5th Omicron wave (Table 2). Waves 1 to 3 were associated with an increased risk of occurrence of ICU admission or in-hospital death in comparison with wave 5: aOR 1.29 [95% CI 1.12 to 1.49] for wave 1, aOR 1.25 [95% CI 1.08 to 1.45] for wave 2 and aOR 1.56 [95% CI 1.36 to 1.79] for wave 3, in multivariate analysis (Table 2 and Table S5).

### Other outcomes

Overall occurrence of respiratory failure was 4,977 (25%), 315 (15%) occurring during the 5th Omicron wave. Occurrence of respiratory failure was significantly less frequent during the 5th Omicron wave than during waves 1 to 4 (Table 2).

Other outcomes are detailed in Table 2. Overall occurrence of MACE was 836 (4%), 128 (6%) being during wave 5, significantly more frequently than during waves 1 to 3. Major bleeding occurred in 1,894 patients (9%) in the overall population, 247 (12%) during wave 5, significantly more frequently than during waves 1 to 3. Overall occurrence of thromboembolic diseases was 1,006 (5%), 88 (4%) during wave 5, significantly less frequently than during waves 3 and 4.

## Discussion

This study reports a lower in-hospital mortality risk of older adults hospitalized with COVID-19 during the 5th Omicron wave, in comparison with waves 1 and 3, after adjusting for confounding factors. These results were confirmed using sensitivity analysis according to variant type. Risk of ICU admission or in-hospital mortality was also lower during the 5th Omicron wave, comparatively with waves 1 to 3. In-hospital mortality remained however high (17%) during the 5th Omicron wave.

Our results are consistent with previously published studies. A multicenter South African study also found a decreased in-hospital mortality during the Omicron surge, decreasing from 29% in the previous wave to 3% [9]. Another nationwide South African study found that S-gene target failure positivity (used as a proxy marker for Omicron infections) was associated with 70% lower odds of severe disease (defined as occurrence of death, respiratory distress syndrome, admission to an ICU, use of any kind of oxygenation and ventilation) in comparison with the Delta variant [2]. In France, infection with the Omicron variant in patients consulting at the emergency department was associated with better in-hospital outcomes, compared to those infected with the Delta variant [7]. The reasons for this lower severity are unclear and probably multifactorial. It is possible, although unconfirmed, that the Omicron variant has a lower intrinsic virulence. Another possibility is that patients during the 5th Omicron wave were more immune to SARS-CoV-2, due to previous infections and/or vaccinations [17–19]. At the beginning of the 5th wave, 98% and 87% of the French population of 75–80 years and ≥ 80 years had received at least one SARS-CoV-2 vaccine dose [8]. On the other hand, although vaccination of the French population aged ≥ 75 years started in January 2021, we still found a high mortality rate during the 4th wave (26%), where the vaccination rate with at least one dose was 93% and 87% among patients aged 75–80 and ≥ 80 years [8]. We have also included in the analysis only the first hospitalization in AP-HP, thus decreasing the risk of having patients with re-infections, although we cannot exclude that patients experienced previous infections without requiring hospitalization. Finally, these patients have already survived previous waves.

This study reports a persistently high in-hospital mortality during the 5th Omicron wave, reaching 17%. Our findings differ from previous studies on the Omicron variant: one performed in South Africa (median age 36 to 59 years) and one in France (median age 58 years) with a mortality rate of 3 and 11% respectively [7, 9]. Our study reports that patients of the 5th Omicron wave had less respiratory failures than during the previous waves, therefore suggesting that virulence of the variant was less important, which cannot explain this high mortality rate. Charlson score was similar in all waves, with a median of 3, suggesting that comorbidity alone could not explain either this high mortality rate during the 5th Omicron wave. The role of age alone is not plausible either, as this 17% in-hospital mortality found during the 5th Omicron wave is higher than what is usually described in acute geriatric wards, usually reaching 11% [20, 21]. One hypothesis could be that a combination of age, comorbidities, functional ability, SARS-CoV-2 infection and COVID-19 management could explain this persistent excess mortality. For example, this high mortality rate could in part be due to an unclear benefit/risk balance of COVID-19 treatments, as older patients are often under-represented in clinical trials [22–26]. Between one quarter to a third of patients from our cohort were treated with glucocorticoids, although adverse events are known to be frequent, particularly in the older population [27–29]. The sub-group analysis of patients aged > 70 years in the RECOVERY trial has not found a significant decrease of 28-day mortality with glucocorticoid treatment, but it is possible that there was a lack of power in this subgroup [22]. In addition, a multicenter retrospective French cohort study in 15 acute COVID-19 geriatric wards during waves 1 to 3 (n = 1,579 patients) found that the use of glucocorticoids was associated with an increased in-hospital mortality [13]. Therefore, the benefit of glucocorticoids remains questionable, since it has still not been specifically tested in the older population. Validation of COVID-19 treatments in this population remains an unmet need.

This study is the first large multicenter cohort of older patients hospitalized with COVID-19 across the 5 waves, population that is often under-represented in studies. The AP-HP network accounts for a large population of hospitalized patients in the Greater Paris Area, representing 10% of all hospitalizations in France, and was in front line for treating COVID-19 patients in all waves. The entire acute care pathway was analyzed, from acute medical wards to ICUs, thus limiting under-estimation of mortality. Inclusion of patients ended 2,5 months before data extraction, therefore limiting under-estimation of mortality during the 5th Omicron wave [30].

The study’s limitations include the presence of potential residual confounding factors and the absence of data on vaccinal status and previous SARS-CoV-2 infections. The presence of the Omicron variant during the 4th wave could explain the absence of difference in mortality between waves 4 and 5. The small number of patients included in wave 2 could have decreased the power of the study, possibly explaining the absence of significant difference of mortality between waves 2 and 5. The differences in wave lengths could impact mortality between the waves. The AP-HP network has patients from the Greater Paris Area, and extrapolation to other regions and countries with different vaccine coverage and health systems can thus be limited. The database being based on medical records, information on the dwelling place, functional ability, or disease duration before hospitalization are lacking. To finish, the cause of death was not available, we could thus not determine the direct responsibility of COVID-19 on death.

Our results show that progress awaits to be made in management of older patients with COVID-19. First, clinical trials including older patients should be performed to confirm the benefit/risk balance of COVID-19 treatments. Second, progress on management of comorbidities and prevention of loss of functional ability should be made. Third, efforts should continue to propose a broader vaccinal coverage, notably for older isolated patients.

## Conclusion

To conclude, mortality was lower during the 5th Omicron wave in the older population, but remained high. These results imply that this variant could be considered as “milder” than the previous variants but not “mild” among patients with advanced age, comorbidities, loss of functional ability and possible iatrogenic events [31]. Efforts should focus on proposing a broader vaccinal coverage, optimizing management of comorbidities and iatrogenic events. There is an unmet need for clinical trials including older patients to confirm the benefit/risk balance of COVID-19 treatments in this fragile population.

### Electronic supplementary material

Below is the link to the electronic supplementary material.

Supplementary Material 1

## Data Availability

Data supporting this study can be made available on request (secretariat.cse@aphp.fr or Claire Hassenkhodja claire.hassenkhodja@aphp.fr) on condition that the research project is accepted by The Clinical Data Warehouse of Greater Paris University Hospitals’ Scientific and Ethics Committee.

## List of Abbreviations

aOR: Adjusted odds ratios
AP-HP: Assistance Publique Hôpitaux de Paris
ATC: Anatomical Therapeutic Chemical
COVID-19: Coronavirus disease 19
CI: Confidence interval
CRP: C-reactive protein
ICD-10: International Classification of Diseases 10th edition
ICU: Intensive care unit
IQR: Interquartile range
MACE: Major adverse cardiovascular events
RECORD: REporting of studies Conducted using Observational Routinely collected health Data
SARS-CoV-2: Severe acute respiratory syndrome coronavirus 2
