# Supplementary material for: In-hospital mortality of older patients with COVID-19 throughout the epidemic waves in the great Paris area: a multicenter cohort study
Source: BMC Geriatr. 2023 Sep 18;23:573. doi: 10.1186/s12877-023-04236-y (PMC10507910; doi:10.1186/s12877-023-04236-y)
Supplement: Supplementary file 1 — Supplementary Material 1 [file 12877_2023_4236_MOESM1_ESM.docx]

**Supplementary file**

**Table S1:** The RECORD statement – checklist of items, extended from the STROBE statement, that should be reported in observational studies using routinely collected health data. **Pages 2-6.**

**Table S2:** Definitions used to identify comorbid conditions, complications and drugs. **Page 7.**

**Table S3:** Characteristics of older patients admitted with a SARS-CoV-2 infection according to variants. **Page 8.**

**Table S4:** Multivariate analysis of in-hospital mortality risk in hospitalized older patients with COVID-19 (according to variant type). **Page 9.**

**Table S5:** Multivariate analysis of risk of in-hospital mortality or admission in intensive care unit in hospitalized older patients with COVID-19 (according to wave number). **Page 10.**

**Table S1:** The RECORD statement – checklist of items, extended from the STROBE statement, that should be reported in observational studies using routinely collected health data.

|  | **Item No.** | **STROBE items** | **Location in manuscript where items are reported** | **RECORD items** | **Location in manuscript where items are reported** |
| --- | --- | --- | --- | --- | --- |
| **Title and abstract** | | | | | |
|  | 1 | (a) Indicate the study’s design with a commonly used term in the title or the abstract  (b) Provide in the abstract an informative and balanced summary of what was done and what was found | Abstract, page 3  Abstract, page 3 | RECORD 1.1: The type of data used should be specified in the title or abstract. When possible, the name of the databases used should be included.  RECORD 1.2: If applicable, the geographic region and timeframe within which the study took place should be reported in the title or abstract.  RECORD 1.3: If linkage between databases was conducted for the study, this should be clearly stated in the title or abstract. | Abstract, page 3  Abstract, page 3  No linkage |
| **Introduction** | | | | | |
| Background rationale | 2 | Explain the scientific background and rationale for the investigation being reported | Introduction, Page 4 |  |  |
| Objectives | 3 | State specific objectives, including any prespecified hypotheses | Introduction, Page 4 |  |  |
| **Methods** | | | | | |
| Study Design | 4 | Present key elements of study design early in the paper | Methods, Page 5 |  |  |
| Setting | 5 | Describe the setting, locations, and relevant dates, including periods of recruitment, exposure, follow-up, and data collection | Methods, Pages 5-6 |  |  |
| Participants | 6 | *(a) Cohort study* - Give the eligibility criteria, and the sources and methods of selection of participants. Describe methods of follow-up  *(b) Cohort study* - For matched studies, give matching criteria and number of exposed and unexposed | Methods, Page 6  No matching | RECORD 6.1: The methods of study population selection (such as codes or algorithms used to identify subjects) should be listed in detail. If this is not possible, an explanation should be provided.  RECORD 6.2: Any validation studies of the codes or algorithms used to select the population should be referenced. If validation was conducted for this study and not published elsewhere, detailed methods and results should be provided.  RECORD 6.3: If the study involved linkage of databases, consider use of a flow diagram or other graphical display to demonstrate the data linkage process, including the number of individuals with linked data at each stage. | Methods, Page 6  eTable 2  Methods, Page 5-6  No linkage |
| Variables | 7 | Clearly define all outcomes, exposures, predictors, potential confounders, and effect modifiers. Give diagnostic criteria, if applicable. | Methods, Pages 6-7 | RECORD 7.1: A complete list of codes and algorithms used to classify exposures, outcomes, confounders, and effect modifiers should be provided. If these cannot be reported, an explanation should be provided. | Methods, Pages 6-7  eTable 2 |
| Data sources/ measurement | 8 | For each variable of interest, give sources of data and details of methods of assessment (measurement).  Describe comparability of assessment methods if there is more than one group | Methods, Page 5 |  |  |
| Bias | 9 | Describe any efforts to address potential sources of bias | Methods, Pages 7-8 |  |  |
| Study size | 10 | Explain how the study size was arrived at | Methods, Pages 5-6, Figure 1 |  |  |
| Quantitative variables | 11 | Explain how quantitative variables were handled in the analyses. If applicable, describe which groupings were chosen, and why | Methods , page 7 |  |  |
| Statistical methods | 12 | (a) Describe all statistical methods, including those used to control for confounding  (b) Describe any methods used to examine subgroups and interactions  (c) Explain how missing data were addressed  (d) *Cohort study* - If applicable, explain how loss to follow-up was addressed  (e) Describe any sensitivity analyses | Methods , page 7-8 |  |  |
| Data access and cleaning methods |  | .. |  | RECORD 12.1: Authors should describe the extent to which the investigators had access to the database population used to create the study population.  RECORD 12.2: Authors should provide information on the data cleaning methods used in the study. | Methods, Pages 7-8  Acknowledgments, page 13  Methods, Page 7-8 |
| Linkage |  | .. |  | RECORD 12.3: State whether the study included person-level, institutional-level, or other data linkage across two or more databases. The methods of linkage and methods of linkage quality evaluation should be provided. | No linkage |
| **Results** | | | | | |
| Participants | 13 | (a) Report the numbers of individuals at each stage of the study (*e.g.*, numbers potentially eligible, examined for eligibility, confirmed eligible, included in the study, completing follow-up, and analysed)  (b) Give reasons for non-participation at each stage.  (c) Consider use of a flow diagram | Results, Page 8; Figure 1  Figure 1  Figure 1 | RECORD 13.1: Describe in detail the selection of the persons included in the study (*i.e.,* study population selection) including filtering based on data quality, data availability and linkage. The selection of included persons can be described in the text and/or by means of the study flow diagram. | Results, Page 8; Figure 1 |
| Descriptive data | 14 | (a) Give characteristics of study participants (*e.g.*, demographic, clinical, social) and information on exposures and potential confounders  (b) Indicate the number of participants with missing data for each variable of interest  (c) *Cohort study* - summarise follow-up time (*e.g.*, average and total amount) | Results, Page 8; Table 1 |  |  |
| Outcome data | 15 | *Cohort study* - Report numbers of outcome events or summary measures over time | Results, Page 8  Table 2 |  |  |
| Main results | 16 | (a) Give unadjusted estimates and, if applicable, confounder-adjusted estimates and their precision (e.g., 95% confidence interval). Make clear which confounders were adjusted for and why they were included  (b) Report category boundaries when continuous variables were categorized  (c) If relevant, consider translating estimates of relative risk into absolute risk for a meaningful time period | Results, Pages 8-9  Tables 2 and 3 |  |  |
| Other analyses | 17 | Report other analyses done—e.g., analyses of subgroups and interactions, and sensitivity analyses | Results, Pages 8-9  Table 2 |  |  |
| **Discussion** | | | | | |
| Key results | 18 | Summarise key results with reference to study objectives | Discussion Pages 9-10 |  |  |
| Limitations | 19 | Discuss limitations of the study, taking into account sources of potential bias or imprecision. Discuss both direction and magnitude of any potential bias | Discussion Page 12 | RECORD 19.1: Discuss the implications of using data that were not created or collected to answer the specific research question(s). Include discussion of misclassification bias, unmeasured confounding, missing data, and changing eligibility over time, as they pertain to the study being reported. | Discussion Page 12 |
| Interpretation | 20 | Give a cautious overall interpretation of results considering objectives, limitations, multiplicity of analyses, results from similar studies, and other relevant evidence | Discussion Pages 9-12 |  |  |
| Generalisability | 21 | Discuss the generalisability (external validity) of the study results | Discussion Page 9-11 |  |  |
| **Other Information** | | | | | |
| Funding | 22 | Give the source of funding and the role of the funders for the present study and, if applicable, for the original study on which the present article is based | Page 13 |  |  |
| Accessibility of protocol, raw data, and programming code |  | .. |  | RECORD 22.1: Authors should provide information on how to access any supplemental information such as the study protocol, raw data, or programming code. | Acknowledgments, page 13 |

*Notes*: *Reference: Benchimol EI, Smeeth L, Guttmann A, Harron K, Moher D, Petersen I, Sørensen HT, von Elm E, Langan SM, the RECORD Working Committee. The REporting of studies Conducted using Observational Routinely-collected health Data (RECORD) Statement. *PLoS Medicine* 2015; in press.

*Checklist is protected under Creative Commons Attribution ([CC BY](http://creativecommons.org/licenses/by/4.0/)) license.

**Table S2:** Definitions used to identify comorbid conditions, complications and drugs

| **Comorbidities at baseline** | **Hospital discharge diagnoses (ICD-10 codes)** |
| --- | --- |
| **Covid-19** | U07.1 except U0713, U10.9 |
| **Comorbidities at baseline** | |
| Organ transplantation | Z94 |
| Systematic auto-immune disease | K50, K51, M05-M09, M45, M46, L93, L94, M30-M36 |
| Any tumor (including lymphoma and leukemia) | D00–D09, C00–C26, C30–C41, C43–C58, C60–C97, D37–D48 |
| Non-valvular atrial fibrillation | I48 |
| Coronary heart disease | I20-I25 |
| Diabetes | E10–E14  G590, G632, G730, G990, H280, H360, I792, L97, M142, M146, N083, G590, G632, G730, G990, H280, H360, I792, L97, M142, M146, N083, T383, Y423 |
| Hypertension | I10, I11, I12, I13, I15 |
| Chronic kidney disease | N18;I12.0; I13.1; I13.2; E10.2; E11.2; E13.2; E14.2? N08.3; Z49.0-Z49.2; Z94.0; Z99.2 |
| Dementia | F00; F01; F02; F03; F05.1; G30; G31.1 |
| COPD | J43; J44; |
| Heart failure | I11.0; I13.0; I13.2; I13.9; I50; K76.1; J81 |
| **Complications during hospitalization** | **Hospital discharge diagnoses (ICD-10 codes)** |
| Respiratory failure | J96.0, J80 |
| Venous thrombo-embolism | I26; I80 (except I80.0); I81; I82 |
| Major bleeding | I60-I62; S063; S064; S065; S066; K250; K252; K254; K256; K260; K262; K264; K266; K270; K272; K274; K276; K280; K282; K284; K286; K290; K920; K921; K922; I850; N02; R31; J942; R040; R041; R042; R048; R049; D62; K661; K624; M250; R58; N920; N921; N924; N938; N939; N950; H113; H356; H431; H450; H922; I312  + Transfusion: Z513 |
| MACE | I63 (except I63.6); G46 related to I63 or I69.3; I74; G45; I21; I22; I24 |
| **Therapeutics** | **ATC codes** |
| Tocilizumab | L04AC07 |
| Glucocorticoids | H02AB |
| Invasive ventilation | GLLD004, GLLD006, GLLD008, GLLD013, GLLD015 |

Abbreviations: ATC: Anatomical Therapeutic Chemical classification system, COPD: Chronic obstructive pulmonary disease, MACE (major adverse cardiovascular events): stroke, myocardial infarction, systemic arterial embolism

**Table S3:** Characteristics of older patients admitted with a SARS-CoV-2 infection according to variants

|  | **ALPHA ^a^**  **N = 1502** | **BETA ^a^**  **N = 96** | **DELTA ^a^**  **N = 777** | **OMICRON ^a^**  **N = 964** | ***Global P-value^b^*** |
| --- | --- | --- | --- | --- | --- |
| **Age, median [IQR]** | 84 [79-89]* | 84 [80-90] | 84 [79-89]* | 85 [80-90] | **0.02** |
| **Female sex, n (%)** | 761 (51) | 50 (52) | 375 (48) | 489 (51) | 0.67 |
| **Comorbidities at baseline, n (%)** | | | | | |
| Dementia | 374 (25)* | 31 (32) | 203 (26)* | 332 (34) | **<0.001** |
| Atrial fibrillation | 395 (26)* | 27 (28) | 198 (25)* | 307 (32) | **0.009** |
| Diabetes | 1060 (29) | 27 (28) | 229 (29) | 275 (29) | 0.95 |
| Hypertension | 834 (56) | 56 (58) | 436 (56) | 565 (59) | 0.48 |
| Chronic kidney disease | 380 (25) | 31 (32) | 168 (22)* | 276 (29) | **0.004** |
| Coronary artery disease | 236 (16)* | 12 (12) | 148 (19)* | 218 (23) | **<0.001** |
| Heart failure | 402 (27)* | 28 (29) | 185 (24)* | 315 (33) | **0.004** |
| COPD | 180 (12)* | 7 (7)* | 106 (14)* | 172 (18) | **<0.001** |
| Any tumor (including lymphoma and leukemia) | 332 (22)* | 17 (18)* | 169 (22)* | 285 (30) | **<0.001** |
| Organ transplantation | 21 (1) | 1 (1) | 12 (2) | 17 (2) | 0.91 |
| Systemic auto-immune diseases | 65 (4)* | 6 (6) | 42 (5)* | 68 (7) | **0.003** |
| Charlson Index median (IQR)  *Missing values* | 3 [1 – 5]*  *362* | 3 [1 – 5]  *22* | 3 [1 – 5]*  *191* | 3 [2 – 5]  *130* | **<0.001** |
| **Biological data at baseline, n (%)^c^** | | | | | |
| Lymphocyte count at baseline <0.81 10^9^/L  *Missing values* | 690 (47)*  *30* | 53 (56)*  *1* | 344 (48)*  *66* | 338 (40)  *124* | **<0.001** |
| CRP at baseline ≥65 mg/L  *Missing values* | 664 (46)*  *46* | 47 (50)*  *3* | 337 (48)*  *79* | 300 (36)  *142* | **<0.001** |
| **Treatments, n (%)** |  |  |  |  |  |
| Glucocorticoids | 635 (42)* | 45 (47)* | 343 (44)* | 296 (31) | **< 0.001** |
| Tocilizumab | 81 (5)* | 3 (3) | 89 (11)* | 27 (3) | **< 0.001** |

*Notes*: ^a^ 1st wave between March 1st 2020 and July 31st 2020, 2nd wave between August 1st 2020 and December 31st 2020, 3rd wave between January 1st 2021 and June 30th 2021, 4th wave between July 1st 2021 and December 31st 2021, and 5th wave between January 1st 2022 and January 31st 2022.

^b^ Chi-squared test or Fisher’s exact test was used for categorical variables and Kruskal Wallis test was used for continuous variables. ^c^ Continuous variables were dichotomized by receiver operating characteristic curve analysis to determine the best threshold for in-hospital mortality (maximization of the Youden index). * *P* value < 0.05 versus Omicron variant (reference).

Abbreviations: COPD: Chronic obstructive pulmonary disease, CRP: C-reactive protein; IQR: interquartile range; NA: not available. Missing values are detailed only when they exist. All codes for comorbidities and treatments can be found in the supplement.

**Table S4:** Multivariate analysis of in-hospital mortality risk in hospitalized older patients with COVID-19 (according to variant type)

| **Variables** | **OR (95% CI)^a^** | ***P* value** |
| --- | --- | --- |
| **Sex, reference value = Female**  Gender = Male | 1.42 (1.19 – 1.69) | **<0.001** |
| **Age, reference value = 75 – 84 years**  Age: 85- 94  Age: ≥ 95 | 1.51 (1.01 – 1.46)  1.29 (0.92 – 1.82) | **0.04**  0.6 |
| **COVID-19 variants, reference value = Omicron**  Alpha  Beta  Delta | 1.90 (1.52 – 2.38)  2.10 (1.28 – 3.38)  1.77 (1.37 – 2.29) | **<0.001**  **0.003**  **<0.001** |
| **Coronary heart disease, reference value = no**  Coronary heart disease = yes | 8.81 (0.64 – 1.01) | 0.71 |
| **Non-valvular atrial fibrillation, reference value = no**  Non-valvular atrial fibrillation = yes | 1.30 (1.71 – 1.57) | **0.007** |
| **Organ transplantation, reference value = no**  Organ transplantation = yes | 0.88 (0.43 – 1.69) | 0.72 |
| **Tumor, reference value = no**  Tumor = yes | 1.05 (0.86 – 1.28) | 0.59 |
| **Chronic kidney disease, reference value = no**  Chronic kidney disease = yes | 1.24 (1.01 – 1.53) | **0.04** |
| **Dementia, reference value = no**  Dementia = yes | 0.77 (0.63 – 0.95) | **0.01** |
| **COPD, reference value = no**  COPD = yes | 0.92 (0.72 – 1.19) | 0.55 |
| **Diabetes, reference value = no**  Diabetes = yes | 1.04 (0.86 – 1.26) | 0.67 |
| **CRP at baseline, reference value < 65 mg/L**  CRP at baseline ≥ 65 mg/L = yes | 2.14 (1.80 – 2.55) | **<0.001** |
| **Lymphocytes count at baseline, reference value > 0.81 10^9^/L** |  |  |
| Lymphocyte count at baseline < 0.81 10^9^/L = yes | 1.26 (1.07 – 1.49) | **0.007** |

*Notes*: ^a^ N = 3,048; C-index 0.66 [95% CI 0.64 to 0.68]; AIC 3,323.

Abbreviations: COPD: Chronic obstructive pulmonary disease, CRP: C-reactive protein, OR, odds ratio; CI, confidence interval.

**Table S5:** Multivariate analysis of risk of in-hospital mortality or admission in intensive care unit in hospitalized older patients with COVID-19 (according to wave number)

| **Variables** | **OR (95% CI)^a^** | ***P-*value** |
| --- | --- | --- |
| **Sex, reference value = Female**  Gender = Male | 1.41 (1.31 – 1.53) | **<0.001** |
| **Age, reference value = 75 – 84**  Age: 85- 94  Age: ≥ 95 | 0.84 (0.78 – 0.91)  0.77 (0.66 – 0.90) | **<0.001**  **<0.001** |
| **COVID-19 waves, reference value = wave** 5  Wave 1  Wave 2  Wave 3  Wave 4 | 1.29 (1.12 – 1.49)  1.25 (1.08 – 1.45)  1.56 (1.36 – 1.79)  1.17 (0.98 – 1.38) | **<0.001**  **0.002**  **<0.001**  0.08 |
| **Coronary heart disease, reference value = no**  Coronary heart disease = yes | 1.17 (1.06 – 1.29) | **0.008** |
| **Non-valvular atrial fibrillation, reference value = no**  Non-valvular atrial fibrillation = yes | 1.31 (1.20 – 1.43) | **<0.001** |
| **Organ transplantation, reference value = no**  Organ transplantation = yes | 1.42 (1.02 – 1.95) | **0.003** |
| **Tumor, reference value = no**  Tumor = yes | 1.03 (0.94 – 1.11) | 0.5 |
| **Chronic kidney disease, reference value = no**  Chronic kidney disease = yes | 1.07 (0.98 – 1.17) | 0.2 |
| **Dementia, reference value = no**  Dementia = yes | 0.65 (0.60 – 0.71) | **<0.001** |
| **COPD, reference value = no**  COPD = yes | 0.99 (0.89 – 1.10) | 0.4 |
| **Diabetes, reference value = no**  Diabetes = yes | 1.04 (0.96 – 1.13) | 0.8 |
| **CRP at baseline, reference value <65 mg/L**  CRP at baseline ≥65 mg/L = yes | 2.19 (2.03 – 2.36) | **<0.001** |
| **Lymphocytes count at baseline, reference value ≥0.81 10^9^/L** | |  |
| Lymphocyte count at baseline <0.81 10^9^/L = yes | 1.47 (1.36 – 1.58) | **<0.001** |

*Notes*: ^a^ N = 14,108; C-index 0.67 [95% CI 0.66 to 0.68]; AIC: 16,724.

Abbreviations: COPD: Chronic obstructive pulmonary disease, CRP: C-reactive protein, OR, odds ratio; CI, confidence interval.
